# Draft Genome Sequence of *Thermoanaerobacter* sp. Strain YS13, a Novel Thermophilic Bacterium

**DOI:** 10.1128/genomeA.00584-15

**Published:** 2015-06-04

**Authors:** Tingting Peng, Siyi Pan, Lew Christopher, Richard Sparling, David B. Levin

**Affiliations:** a Department of Food Science, Huazhong Agricultural University, Wuhan, China; b Biorefining Research Institute (BRI), Lakehead University, Thunder Bay, Ontario, Canada; c Department of Microbiology, University of Manitoba, Winnipeg, Manitoba, Canada; d Department of Biosystems Engineering, University of Manitoba, Winnipeg, Manitoba, Canada

## Abstract

Here, we report the draft genome sequence of *Thermoanerobacter* sp. YS13, isolated from a geothermal hot spring in Yellowstone National Park, which consists of 2,713,030 bp with a mean G+C content of 34.05%. A total of 2,779 genes, including 2,707 protein-coding genes, 12 rRNAs, and 59 tRNAs were identified.

## GENOME ANNOUNCEMENT

*Clostridium*, *Thermoanaerobacterium*, and *Thermoanaerobacter* are genera that contain a diverse group of anaerobic, thermophilic, and lignocellulolytic bacteria (1, 2). *Thermoanaerobacter* sp. YS13 is a strictly anaerobic thermophilic bacterium isolated from a geothermal hot spring in Yellowstone National Park. Phylogenetic analysis using a 16S rRNA gene sequence suggested strain YS13 as a species of *Thermoanaerobacter* that is able to grow over a wide range of temperatures and pHs and synthesizes hydrogen (H_2_), carbon dioxide (CO_2_), ethanol, acetate, and lactate as the dominant fermentation end products using a wide range of carbohydrate substrates (3).

The genome of *Thermoanaerobacter* sp. YS13 was sequenced at the McGill University Genome Quebec Innovation Centre using a PacBio RSII sequencer, which generated a total of 77,846 raw subreads with an average length of 4,073 bp. Genome assembly was performed by the HGAP workflow (4). The raw subreads were first preassembled using BLASR (5) to align short subreads on long subreads to correct sequencing errors before assembly. The long corrected reads were used to assemble the genome using a Celera Assembler (6). After Celera assembly, the raw reads on contigs were realigned by BLASR, and then processed by Quiver to generate high-quality consensus sequences. The assembled draft genome consists of 5 contigs (size >2,638 bp) with a total size of 2,713,030 bp, an *N*_50_ contig length of 1,514,408 nucleotides, and a mean G+C content of 34.05%.

The assembled genome was submitted to the Joint Genome Institute’s (JGI) Integrated Microbial Genomes-Expert Review (IMG ER) platform (7) and annotated using their annotation pipeline. The annotated genome sequences were submitted to the JGI Gene Prediction Improvement Pipeline (GenePRIMP) (8) and were further subjected to manual curation using Artemis (9). The graft genome sequence of *Thermoanarobacter* sp. YS13 is estimated to have a total of 2,779 genes, including 2,707 protein-coding genes, 59 tRNAs, and 12 rRNAs.

### Nucleotide sequence accession numbers.

The genome sequence of *Thermoanaerobacter* sp. YS13 has been deposited at DDBJ/EMBL/GenBank under the accession number JOOI00000000. The version described in this paper is the first version, JOOI01000000.
